# From Brain Organoids to Translational Neurology: Exploring Neuroprotective Targets and Molecular Approaches in Perinatal Brain Injury

**DOI:** 10.3390/cells15050462

**Published:** 2026-03-04

**Authors:** Anja Harej Hrkać, Ana Pelčić, Silvestar Mežnarić, Jasenka Mršić-Pelčić, Kristina Pilipović

**Affiliations:** 1 Department of Basic and Clinical Pharmacology and Toxicology, Faculty of Medicine, University of Rijeka, 51000 Rijeka, Croatia; aharej@medri.uniri.hr (A.H.H.); silvestar.meznaric@medri.uniri.hr (S.M.); jasenka.mrsic.pelcic@medri.uniri.hr (J.M.-P.); 2 University Hospital Centre Rijeka, 51000 Rijeka, Croatia; ana.pelcic@gmail.com

**Keywords:** brain organoids, hypoxic–ischemic injury, neuroinflammation, PBI, pharmacogenomics, precision medicine

## Abstract

Perinatal brain injury (PBI) is a leading cause of long-term neurological deficits in newborns, yet effective therapies are limited. At the cellular level, PBI involves hypoxic–ischemic stress, neuroinflammation, oxidative damage, excitotoxicity, and disrupted neurovascular and glial development. Traditional animal models and 2D cultures cannot fully capture the spatiotemporal complexity of the developing human brain, highlighting the need for more physiologically relevant systems. Human brain organoids have emerged as advanced three-dimensional models that recapitulate region-specific cytoarchitecture, neuronal and glial differentiation, and early circuit formation. They enable modeling of hypoxic–ischemic and inflammatory insults, allowing for the study of injury-induced changes in neurogenesis, gliogenesis, synaptic development, and cell interactions. Organoids facilitate identification of molecular pathways involved in injury and repair, supporting therapeutic target discovery. Using patient-derived induced pluripotent stem cells, organoids also allow personalized pharmacogenomic studies to assess genotype-dependent drug responses and toxicity. Despite limitations such as variability, lack of vascularization and immune components, and ethical considerations, brain organoids offer a promising platform to bridge developmental neurobiology and translational therapeutics, paving the way for targeted and individualized interventions in PBI.

## 1. Introduction

Perinatal brain injury (PBI) remains one of the most devastating challenges in neonatal medicine and developmental neuroscience, representing a leading cause of neonatal mortality and lifelong neurological disability worldwide. In most cases, a common pathway of injury is elicited by an initial hypoxic–ischemic or inflammatory insult, initiating a cascade of events that potentiates perinatal brain injury [1,2]. PBI can affect infants born at any gestational age; however, very preterm fetuses (born <32 weeks of gestation) are less equipped to adapt to perinatal insults than term infants, making them more prone to brain injury. The global incidence of PBI remains alarmingly high, affecting approximately 9.9% of births worldwide [1]. Despite significant advances in perinatal monitoring and intensive care, the overall burden of PBI has not substantially declined, highlighting a persistent gap between clinical need and effective neuroprotective strategies. Therapeutic hypothermia (TH) was the first empirically supported neuroprotective treatment for neonates with hypoxic–ischemic encephalopathy (HIE) and has become the clinical standard of care [2]. Although therapeutic hypothermia (TH) has demonstrated proven efficacy, its overall effectiveness remains limited. Many clinical trials continue to report high mortality rates, significant disability, and persistent neurological complications among newborns. Evidence suggests that combining TH with pharmacological treatment may offer improved outcomes in managing hypoxic–ischemic encephalopathy (HIE). A meta-analysis conducted at the University of Toronto in Canada found that the addition of neuroprotective agents shortened hospital stays for newborns with HIE compared to TH alone. However, these adjunct therapies did not significantly reduce the risk of death or long-term neurological impairment. The agents evaluated included phenobarbital, topiramate, magnesium sulfate, melatonin, erythropoietin (EPO), xenon, and stem cells. No single therapy demonstrated clear superiority over the others in terms of effectiveness [3,4,5]. As survival rates of extremely preterm infants continue to improve, the prevalence of neurodevelopmental morbidity has increased, imposing profound personal, societal and economic costs [6]. These realities highlight an urgent need for improved mechanistic insight and the development of novel, developmentally appropriate therapeutic approaches.

At the cellular and molecular levels, PBI is not a single pathological event but a multiphasic and multifactorial process. Acute hypoxic–ischemic insults trigger rapid energy failure, glutamate-mediated excitotoxicity, calcium overload, and activation of proteases, resulting in primary neuronal and glial injury. After reperfusion, a latent phase of partial metabolic recovery is often followed by secondary energy failure, characterized by mitochondrial dysfunction, excessive generation of reactive oxygen species, lipid peroxidation, and DNA damage. These processes converge with sustained neuroinflammation, driven by activated microglia and astrocytes releasing pro-inflammatory cytokines such as IL-1β and TNF-α. Over time, tertiary injury mechanisms, including epigenetic reprogramming and maladaptive synaptic remodeling, further entrench long-term neurodevelopmental deficits [7,8].

Crucially, these pathological cascades occur during highly sensitive periods of brain development. Neural progenitor cells, pre-oligodendrocytes, microglia, and endothelial cells are actively involved in neurogenesis, gliogenesis, synaptogenesis, and neurovascular maturation. Disruption of these tightly regulated processes can permanently alter brain structure and connectivity, even if the initial insult is transient [9,10]. In preterm infants, the selective vulnerability of oligodendrocyte progenitors and developing white matter further worsens long-term functional impairment [11,12]. Understanding how injury interacts with normal human developmental trajectories, therefore, remains a central challenge in PBI research.

Historically, insights into PBI pathophysiology have been derived largely from animal models and two-dimensional (2D) in vitro systems. Rodent and large-animal hypoxia–ischemia models have provided foundational knowledge of excitotoxicity, oxidative stress, and inflammatory signaling, and have enabled preclinical testing of candidate neuroprotective agents [13,14]. However, fundamental species differences in brain size, cortical organization, developmental timing, immune responses, and gene expression limit the translational relevance of these models. Similarly, 2D neural cultures lack the three-dimensional architecture, cellular diversity, biomechanical gradients, and paracrine signaling environments essential for modelling human brain development [15]. These limitations are evident in the high rate of translational failure, with over 90% of neuroprotective agents that show promise in preclinical studies failing in clinical trials [16].

Human brain organoids have emerged as a transformative platform for modelling early human neurodevelopment and disease. The use of brain organoids in PBI research provides unprecedented opportunities to study injury mechanisms within a human-relevant developmental context and provide a powerful platform for identifying dysregulated molecular pathways and therapeutic targets [17]. Moreover, organoids support medium- to high-throughput drug screening, allowing quantitative assessment of neuroprotection, toxicity, and developmental impact in a human-specific context [18,19]. A particularly promising aspect of organoid technology is its application in pharmacogenomics and precision medicine. Patient-derived iPSCs retain individual genetic backgrounds, enabling the generation of organoids that model genotype-dependent susceptibility to injury and variability in treatment response. This is especially relevant for PBI, where genetic modifiers may influence injury severity, repair capacity, and therapeutic efficacy [20,21]. Combined with genome editing approaches such as CRISPR/Cas9, organoids provide a unique system for dissecting gene–environment interactions and testing personalized therapeutic strategies [22]. Despite their transformative potential, brain organoids have limitations, and continued technological innovation, standardized protocols, and ethical oversight are essential to fully realize the translational promise of organoid-based PBI research.

In this review, we explore the emerging role of human brain organoids as advanced models for studying PBI. We examine how these systems enhance understanding of human-specific injury mechanisms, enable identification of novel therapeutic targets, and support the development of precision medicine approaches. By bridging developmental neurobiology and translational neuroscience, brain organoids hold significant promise for reshaping PBI research and improving outcomes for affected infants.

## 2. Developmental Context of Brain Injury: Cellular, Molecular, and Mitochondrial Mechanisms in the Perinatal Period

**Figure 1:**
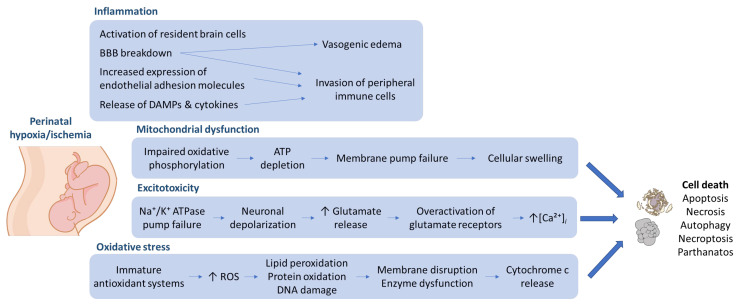
Pathophysiological mechanisms of perinatal hypoxia/ischemia-induced brain injury. Schematic representation of the major mechanisms underlying brain injury following perinatal hypoxia–ischemia. Abbreviations: BBB, blood–brain barrier; DAMPs, danger-associated molecular patterns; ROS, reactive oxygen species.

In the pathophysiology of cerebral hypoxia/ischemia a cascade of cellular events occurs, beginning with the loss of oxygen that causes energy depletion, excitotoxicity, and complex changes in cellular metabolic activity Figure 1 [23]. Early on, post-ischemic inflammation ensues, characterized by involvement of both resident brain cells as well as peripheral immune cells that “invade” the brain through the damaged blood–brain barrier (BBB) [24]. The so-called “sterile” injury of hypoxia/ischemia also involves a massive release of signaling molecules, including danger-associated molecular patterns (DAMPs) and pro-inflammatory cytokines, as well as increased expression of endothelial adhesion molecules that are responsible for peripheral blood cells’ adhesion and brain tissue invasion [25,26,27]. This inflammatory cascade leads to two major complications that often determine a patient’s survival. Due to the BBB breakdown (that starts at around 3–6 h post-ischemia), vasogenic edema develops causing life-threatening swelling of the brain tissue [28]. Secondly, so-called ischemic penumbra (potentially salvageable tissue surrounding the ischemic core) becomes endangered by the toxic inflammatory environment [29].

While the core biological responses to brain injury are similar to the postnatal environment, the perinatal brain (from 20 weeks gestation to 28 days after birth) has several unique characteristics compared to an adult brain [30]. These differences are largely due to the brain’s immaturity, ongoing development, and different immune landscape. These differences can be categorized into cellular vulnerability, the nature of the inflammatory response, and long-term outcomes.

In the perinatal brain, mitochondrial dysfunction functions as a primary driver of injury progression rather than a secondary consequence [31]. Unlike the adult brain, in which mitochondrial failure often reflects terminal dysfunction, the perinatal brain exhibits a distinct, temporally regulated process of mitochondrial deterioration known as secondary energy failure. During the initial hypoxic–ischemic insult (primary energy failure), impaired oxidative phosphorylation leads to ATP depletion, membrane pump failure, and cellular swelling. This is followed by a latent phase lasting during which cerebral metabolism appears stable and represents a critical therapeutic window. Subsequently, secondary energy failure occurs, characterized by progressive mitochondrial dysfunction and renewed ATP depletion despite restored oxygenation, resulting in amplified neuronal injury. In the perinatal brain, mitochondrial dysfunction preferentially activates apoptotic rather than necrotic pathways; neonatal mitochondria exhibit a low threshold for mitochondrial permeability transition pore opening, promoting cytochrome c release and caspase activation [32]. The immature brain is also highly sensitive to apoptosis-inducing factor-mediated, caspase-independent DNA damage. Limited calcium-buffering capacity further predisposes neonatal mitochondria to calcium overload and ROS generation, amplifying intrinsic apoptotic signaling and neuronal loss.

Role of excitotoxicity in post-ischemic injury is important in both perinatal and adult brain [33]. However, in the developing brain, glutamate is not just a neurotransmitter, but also a trophic factor essential for growth, meaning the system is naturally geared toward high excitation. Thus, in the perinatal brain, glutamate receptors are more abundant and function differently to support synaptogenesis and plasticity. Additionally, glutamate clearance in perinatal brain is less active as the expression of glutamate transporters on astrocytes in neonates are significantly lower compared to mature brain, leading to a prolonged excitotoxic insult compared to the sharp spikes of synaptic glutamate levels seen in adults [34].

One of the most significant differences is the manner in which the brain cells die. Namely, while in the adult brain cell death is predominantly necrotic, with massive inflammatory response, in the perinatal brain several forms of regulated cell death (e.g., apoptosis, autophagic cell death/autosis, necroptosis, parthanatos) are predominant [35]. This difference is hypothesized to be due to the fact that, in the perinatal stage, brain is in a state of high “synaptic pruning” with the pathways for apoptosis being highly active. In those circumstances, the neonatal brain is uniquely sensitive to triggers that would not necessarily kill an adult cell [36].

Further on, compared to adults, the neonatal immune system is still very immature, and in the injury response, there are several distinct inflammatory patterns that can be traced [37]. For example, in neonates’ microglial response is more rapid (often within minutes) compared to the hours it takes in adults [38,39]. In adult strokes, neutrophils invade the brain and cause damage, while in the perinatal brain, they often stay trapped in the blood vessels or infiltrate much less significantly [40,41,42], meaning the tissue damage is more significantly related to the internal brain signaling than from peripheral immune invaders. In adults, astrocytes are quick to react and form a tough scar to wall off the injury. Contrarily, in neonates, these glial cells are less reactive, they do not form a scar as effectively, thus allowing the injury to spread or evolve more easily over time [43].

There are also differences in the function of the BBB, beyond the common misconception that the neonatal BBB is immature and leaky. Namely, in the adults’ brain, the BBB is very strong but breaks down violently during a stroke, leading to rapid and massive edema. In the neonates, BBB is much more sensitive to inflammatory signals and even mild inflammation can cause significant increase in BBB permeability, leading to a slower progression of damage compared to the rapid brain swelling seen in adult stroke [44].

Next significant difference is in the selective regional vulnerability as in the perinatal stroke specific regions and cell types are targeted based the stage of the brain development [45]. For example, in preterm infants (24–32 weeks), the most vulnerable area is the periventricular white matter with target cell being pre-oligodendrocytes as these cells are extremely sensitive to free radicals because they lack mature antioxidant defenses [46]. This commonly results in periventricular leukomalacia, which often leads to spastic diplegia. In term infants (37–40 weeks), it is the deep gray matter pattern that is most vulnerable, specifically, the basal ganglia (putamen and globus pallidus) and the thalamus [47]. At term, these areas are the most active parts of the brain with high metabolic demand. Common outcome is dyskinetic cerebral palsy characterized by difficulties in movement and posture control.

Collectively, these mechanisms highlight that PBI is not a just a version of adult neuropathology but a biologically distinct process shaped by developmental stage. The combination of heightened excitability, immature immune signaling, and apoptosis-prone mitochondrial function confers both vulnerability and therapeutic opportunity. Understanding these age-specific mechanisms is essential for designing interventions that target the evolving biology of the perinatal brain rather than extrapolating from adult models.

## 3. Emerging Investigational Therapies: Cell-Based Approaches, Stem Cells, and Extracellular Vesicles

As the current therapy of PBI is limited, there is a significant need for more effective therapeutic approaches, which especially is valid for long-term therapies beyond the acute phase of PBI [48,49]. In recent years, regenerative and reparative strategies have emerged as promising avenues for PBI therapy. Stem cell–based approaches, using mesenchymal stem cells, umbilical cord blood cells, or neural progenitor cells, have shown beneficial effects in preclinical studies, mainly through paracrine mechanisms that modulate inflammation, enhance endogenous repair, promote angiogenesis, and support neurogenesis [50,51].

Stem cells derived from embryonic or fetal tissues, as well as from adult sources such as bone marrow, adipose tissue, and blood, have demonstrated regenerative and anti-inflammatory effects in a range of cardiovascular, pulmonary, and neurological conditions. Interest in cell therapies is also growing in neonatal and pediatric medicine, with ongoing clinical trials exploring their use in disorders such as bronchopulmonary dysplasia (BPD), cerebral palsy (CP), hypoxic–ischemic encephalopathy, and hypoplastic left heart syndrome. These findings indicate that the neuroprotective effects of hAECs in the developing brain are likely driven by their ability to dampen harmful inflammatory responses. Exploring hAECs further as a treatment for preterm brain injury may be an important next step toward understanding their safety and effectiveness in clinical settings [52].

Evidence suggests that the benefits of stem cells-based therapies are primarily mediated through paracrine signaling rather than direct cell replacement. Stem cell-derived extracellular vesicles (SC-EVs) are key mediators of these effects, delivering anti-inflammatory factors, growth factors, and regulatory RNAs.

Extracellular vesicles (EVs) are membrane-bound structures enclosed by a lipid bilayer. Over the past five decades, growing interest in EV biology has led to significant advances in understanding their origins and functions, establishing EVs as promising tools in the treatment of a wide range of diseases. EVs are present in all body fluids and are released by virtually all cell types. Based on their biogenesis and size, EVs are generally classified into exosomes (EXs), microvesicles (MVs), and apoptotic bodies. Microvesicles are larger vesicles that form through outward budding of the plasma membrane. EVs contribute to cellular homeostasis by facilitating waste disposal and communicate with recipient cells through multiple mechanisms, including receptor–ligand interactions, endocytosis, membrane fusion, membrane protein transfer, and the delivery of functional RNAs and proteins [53]. SC-EVs may offer a safer and more effective alternative to stem cells, as they lack tumor-forming capacity, have low immunogenicity, can cross the blood–brain barrier, and can be engineered to deliver therapeutic cargo. These properties make SC-EVs a compelling candidate for future PBI treatment strategies [54].

However, all these approaches remain experimental, with ongoing clinical trials evaluating their safety, feasibility, and efficacy. Significant challenges regarding cell source, dosing, delivery routes, and ethical considerations continue to limit widespread clinical application.

## 4. Human Brain Organoids as Advanced Models of Perinatal Neurodevelopment

Human brain development is a complex, prolonged process orchestrated by both genetic and environmental factors, with maturation of neural circuits and cognitive functions extending over many years. Although genetic factors play a central role in regulating brain developmental programs, the molecular and circuit mechanisms underlying genetically driven neuropsychiatric disorders remain poorly understood. This knowledge gap highlights the need for alternative experimental systems that better reflect human biology [55].

One of the common causes of PBI is hypoxic–ischemic injury specifically that refers to brain damage caused by reduced oxygen supply (hypoxia) and/or reduced blood flow (ischemia) to the brain. It most commonly occurs around the time of birth and is a leading cause of PBI, especially in term infants. After birth, premature infants are at high risk of hypoxia due lung immaturity, hypotension and lack of cerebral-flow regulation. They can develop encephalopathy of prematurity.

Pașca et al. [56] have used human three-dimensional brain-region-specific organoids to study the effect of oxygen deprivation on corticogenesis. They have discovered distinct abnormalities in intermediate progenitors, a cortical cell population critical for human cerebral cortex expansion, and linked these defects to alterations in the unfolded protein response. These findings were further validated in human primary cortical tissue, where they have shown that pharmacological modulation of the unfolded protein response can prevent hypoxia-induced loss of intermediate progenitors. This human-based cellular platform will provide a powerful tool for investigating genetic and environmental mechanisms driving injury in the developing human brain [56].

Perinatal brain injuries that include encephalopathy associated with fetal growth restriction, encephalopathy of prematurity, neonatal encephalopathy in term infants, and neonatal stroke are a leading cause of neurodevelopmental disorders. These conditions initiate complex cellular and molecular cascades that often result in lasting motor, cognitive, and behavioral impairments. The resulting pathology includes neuronal loss, selective vulnerability of interneuron subtypes, impaired maturation of oligodendrocyte progenitor cells with consequent demyelination, axonal injury, and likely synaptic dysfunction, ultimately disrupting neural connectivity. The extent and pattern of damage depend on both the nature and severity of the insult and the developmental stage of the brain.

Microglial activation is a common feature across perinatal brain injuries, positioning these cells as central regulators of both injury progression and repair processes. Their diverse and sometimes opposing roles are driven by dynamic transcriptional states and intricate interactions with other neural cell types. Adding further complexity, microglia undergo distinct developmental stages and actively contribute to normal brain development. Beyond their roles in brain maturation and injury response, microglia also mediate the effects of systemic inflammation on the brain, a major risk factor for adverse neurodevelopmental outcomes in preterm infants. These findings highlight microglia as a critical therapeutic target for neuroprotection in PBI [10].

Because animal models and 2D monolayer cultures cannot fully capture the in vivo complexity of the human central nervous system, three-dimensional brain organoids have emerged as an advanced alternative. Organoid-based platforms provide high experimental and translational value by overcoming key limitations of conventional cell culture systems while remaining compatible with well-established biochemical and cellular analyses traditionally used in 2D cultures. Human brain organoids generated from patient-derived iPSCs, including those obtained from fibroblasts or blood cells, have been widely applied to model a broad spectrum of neurological, developmental, and psychiatric disorders.

By recapitulating patient-specific disease phenotypes, organoid models of microcephaly [57], Seckel syndrome [58], autism [59], Rett syndrome [60], and Miller–Dieker syndrome have substantially advanced our understanding of disease mechanisms. Accordingly, brain organoids that mimic human brain development represent powerful systems for investigating neurodevelopmental disorders. Nevertheless, current organoid models face important limitations, such as cellular immaturity, the lack of certain cell populations (including microglia and vascular cells), and the accumulation of cellular stress, which restrict their ability to fully and accurately model diseases associated with later stages of brain maturation [61].

To date, there are relatively few published scientific studies that have specifically used brain organoids to investigate PBI. Therefore, this approach represents a novel strategy in this complex field.

## 5. Organoid-Based Modeling of Hypoxic–Ischemic and Inflammatory Perinatal Brain Injury

Modeling PBI using organoids represents a significant leap in neonatal neurology [62]. Unlike traditional animal models, human brain organoids allow researchers to study the specific cellular responses of the developing human brain to insults like oxygen deprivation and inflammation [56,63,64]. Organoid models are particularly effective at showing why the preterm brain is so fragile as they allow us to observe damage to specific cell types that are difficult to study in vivo [57].

Existing models for perinatal ischemic injury fall short for various reasons some of those being because in vivo animal models are lissencephalic and lack human-specific white matter complexity, while 2D systems fail to replicate the 3D architecture, metabolic gradients, and intricate cell–cell interactions essential to perinatal pathology. iPSC-derived organoids excel by providing developmental-stage specificity. They can be timed to resemble second-trimester or near-term fetal brains, capturing the peak vulnerability of specific populations like pre-oligodendrocytes and subplate neurons, which are the primary targets in human white matter injury.

By using organoids, researchers have the possibility to also implement “two-hit” experiments to isolate injury mechanisms. However, among the limitations, there is a lack of functional vasculature (leading to necrotic cores) and a missing peripheral immune system, which restricts the study of systemic inflammatory responses and long-term reperfusion effects [65].

### 5.1. Advanced Organoid Models with Enhanced Physiological Relevance

The development of increasingly sophisticated organoid models has been crucial for advancing research and potential therapeutic applications of organoids. Recently developed multi-region brain organoids integrate cerebral, mid/hindbrain, and endothelial systems, providing a more comprehensive model of brain complexity [66]. These assembloids enable investigation of how different brain regions interact during injury and recovery, which is essential for understanding systemic effects of therapeutic interventions. Another major advance toward therapeutic applications lies in the development of vascularized organoid models. Yi and colleagues [67] used single-cell transcriptomics of vascularized human brain organoids to decipher lineage-specific stress adaptation in fetal hypoxia-reoxygenation injury. Same year, Li et al. [68] similarly characterized neural responses to hypoxic injury in vascularized cerebral organoid models, demonstrating improved physiological relevance compared to non-vascularized systems. The presence of functional vasculature is particularly important for modeling drug delivery and studying neurovascular interactions that are central to PBI path.

Daviaud and colleagues [63] used cerebral organoid models to reveal distinct vulnerability and resilience of human neuroprogenitor subtypes in prenatal hypoxic injury. This work highlighted how advanced organoid models can identify cell type-specific therapeutic targets that might be protected through targeted interventions.

### 5.2. Modeling Hypoxic–Ischemic Encephalopathy

In an in vitro laboratory setting, hypoxic–ischemic injury is typically modeled using oxygen-glucose deprivation (OGD). In OGD model, researchers place mature organoids in a hypoxic chamber (usually <1% O_2_) and replace standard media with glucose-free media for a set duration (typically 1 to 6 h), triggering a cascade of events mimicking clinical hypoxia/ischemia, including excitotoxicity, oxidative stress, and structural collapse of the organoid with loss of neural progenitor cell populations [69,70].

Beyond OGD model (still the gold standard for modeling hypoxic–ischemic encephalopathy), researchers have developed several alternative physical and chemical methods to simulate the lack of oxygen and the metabolic failure seen in PBI. By using chemical hypoxia mimetic agents, such as cobalt chloride [71], dimethyloxalylglycine [72], and sodium dithionite [73], rather than physically depleting oxygen, induction of a hypoxia-like state is achieved through stabilizing hypoxia-inducible factor 1-alpha (HIF-1α), the transcription factor that orchestrates cellular responses to oxygen deprivation. By using microfluidics and “organoid-on-a-chip”, much tighter control over the oxygen environment than a simple incubator can be established [74]. These allow making of a gradient of oxygen levels across a single organoid, mimicking the watershed areas of the human brain, i.e., regions at the very end of blood supply lines that are the first to suffer during hypoxic–ischemic injury. With microfluidics, the flow of nutrients to only specific parts of an assembloid can be cut off, simulating a localized stroke or a focal ischemic event rather than a global brain insult.

As neuroinflammation is a significant driver of the post-ischemic secondary injury, there are several ways to introduce external inflammatory triggers to the organoid environment. Most commonly, lipopolysaccharide (LPS), bacterial derivative used to trigger an innate immune response, and various pro-inflammatory cytokines (e.g., TNFα and IL-1β) are used [75]. It is important to note that standard organoids often lack immune cells, including microglia and invading peripheral immune cells [76]. Therefore, to accurately model inflammation, researchers now use assembloids, integrating primitive macrophages or microglia into the brain organoid to see how these immune cells react with developing neurons [74].

## 6. Pharmacogenomic Insights from Brain Organoids in Treatment of Perinatal Brain Injury: Implications for Personalized Therapeutic Targeting

The emergence of human brain organoids derived from iPSCs has opened new possibilities for studying PBI in a more physiologically relevant context [64,67]. Pașca and colleagues [56] established a human 3D cellular model of hypoxic brain injury of prematurity using cortical spheroids, demonstrating that these structures can model key features of injury including cell death patterns and developmental disruption. More recently, vascularized brain organoids have been developed that enable the study of neurovascular interactions during hypoxic injury, which is critical for understanding the full spectrum of pathological changes [67,77,78]. These vascularized models show enhanced physiological relevance by incorporating blood vessel structures and allowing investigation of blood–brain barrier properties [79]. The ability of organoids to model hypoxic injury at single-cell resolution has been particularly valuable. Studies using spinal cord organoids have validated necrotic core-free models for fetal neural ischemia that allow detailed analysis without confounding effects of massive cell death [71]. Kim and colleagues [80] demonstrated modeling of hypoxic brain injury through 3D human neural organoids, showing that these systems reproduce key cellular responses to oxygen deprivation.

### 6.1. Single-Cell Pharmacogenomic Profiling and Therapeutic Target Discovery

One of the most significant advances in organoid-based pharmacogenomics has been the application of single-cell transcriptomics to identify cell type-specific responses to hypoxic injury. Yi and colleagues [67] performed comprehensive single-cell transcriptomic analysis of vascularized human brain organoids subjected to hypoxia-reoxygenation injury, revealing lineage-specific stress adaptation mechanisms. This study identified distinct patterns of vulnerability and adaptive responses across different neural cell types, including developmental arrest in astrocyte precursors and neurogenic collapse in GABAergic neurons. The multi-omics approaches enabled by organoid technology have uncovered novel therapeutic targets that were not apparent from bulk tissue analysis. These include signaling pathways such as Notch signaling, IGF2-IGF2R axis, and LGALS3-MERTK pathways, which show cell type-specific activation patterns during injury and recovery [67,81]. Understanding these lineage-specific vulnerabilities is critical for developing targeted neuroprotective strategies that can protect the most vulnerable cell populations [77].

### 6.2. Drug Screening and Neuroprotective Agent Testing

Brain organoids have emerged as powerful platforms for screening neuroprotective drugs and evaluating their efficacy in human-relevant models. Boisvert and colleagues demonstrated that minocycline, a tetracycline antibiotic with anti-inflammatory properties, could mitigate the effects of neonatal hypoxic insult on human brain organoids [64]. This study provided important validation that organoid systems can identify clinically relevant therapeutic effects. Other studies have explored additional neuroprotective compounds including Integrated Stress Response Inhibitor (ISRIB) and rapamycin, showing region-specific and cell type-specific effects [56,71,80]. The ability to assess drug responses at cellular resolution allows researchers to understand not only whether a drug is effective, but also which cell types benefit most from treatment [82]. This information is crucial for understanding mechanisms of action and predicting clinical efficacy. Organoid-on-chip technologies have further enhanced drug screening capabilities by enabling precise control of microenvironmental conditions and high-throughput analysis [83]. These platforms combine the biological complexity of organoids with the engineering precision of microfluidic systems, allowing for more sophisticated pharmacological studies.

### 6.3. Patient-Derived Organoids and Personalized Medicine Approaches

A major promise of organoid pharmacogenomics lies in the ability to generate patient-specific models that capture inter-individual genetic variation in drug response. Patient-derived organoids can be generated from individual iPSC lines, enabling the assessment of how genetic background influences both injury susceptibility and therapeutic response [77,84,85]. This approach has been successfully implemented in other neurological conditions, with Jacob and colleagues developing a patient-derived glioblastoma organoid biobank that recapitulates inter- and intra-tumoral heterogeneity [86]. More recently, Peng and colleagues demonstrated that individualized patient tumor organoids can faithfully preserve human brain tumor ecosystems and predict patient response to therapy, providing proof of concept for personalized medicine approaches using organoids [87]. Similarly, Wang and colleagues created a phenotypic brain organoid atlas and biobank for neurodevelopmental disorders, demonstrating the feasibility of large-scale organoid-based personalized medicine initiatives [88]. An innovative approach called “Chimeroids,” developed by Antón-Bolaños and colleagues, involves creating organoids from multiple donors to assess individual susceptibility to neurotoxic triggers while controlling for technical variability [89]. This approach could be particularly valuable for understanding genetic factors that influence PBI outcomes.

### 6.4. Integration of Machine Learning and Multi-Omics Data

The complexity of organoid-based pharmacogenomic studies generates vast amounts of data that require sophisticated analytical approaches. Integration of machine learning with organoid platforms has enabled more robust analysis of drug responses and identification of predictive biomarkers [90]. Bruno and colleagues [91] demonstrated label-free detection of biochemical changes during cortical organoid maturation using Raman spectroscopy and machine learning, showing how advanced analytical methods can extract additional information from organoid experiments. Multi-omics platforms that combine transcriptomics, proteomics, and metabolomics data from organoids provide comprehensive molecular profiles of injury and therapeutic responses. Zhu and colleagues developed a stroke organoids-multiomics platform to study injury mechanisms and drug responses, demonstrating the power of integrated approaches [92].

### 6.5. Limitations and Challenges in Organoid Pharmacogenomics, Future Directions and Clinical Translation

Despite remarkable progress, organoid-based pharmacogenomic studies face several important limitations. One major challenge is the limited genetic diversity in most studies, which typically use single-donor iPSC lines [67,83,85]. This limits the generalizability of findings to broader populations and makes it difficult to assess how genetic variation influences drug responses. Bellotti and colleagues [93] discussed these challenges in the context of traumatic brain injury research, emphasizing the need for more diverse organoid biobanks. Translational barriers remain a significant concern, with few organoid-based findings having been validated in clinical settings. Hazra conducted a systematic review on the pharmacogenomic mechanisms of brain organoids, pointing out the gap between preclinical organoid studies and clinical applications [94]. Addressing these limitations will require collaborative efforts between basic researchers, clinicians, and regulatory agencies.

The future of organoid-based pharmacogenomics in PBI research will likely focus on several key areas. First, expanding genetic diversity through large-scale biobanks representing diverse populations will be essential for developing truly personalized therapeutic approaches [85,88]. Second, improving the physiological relevance of organoid models through better vascularization and incorporation of immune components will enhance their predictive value [77,95]. Integration of organoid studies with clinical trials will be crucial for validating organoid-derived insights. This might involve using patient-derived organoids to predict individual treatment responses before initiating therapy, a concept that has shown promise in oncology [87]. Additionally, combining organoid models with other complementary approaches, including animal models and computational modeling, will provide more comprehensive understanding of injury mechanisms and therapeutic strategies. Advances in organoid-on-chip technologies and integration with biosensors will enable real-time monitoring of organoid responses to injury and treatment, providing dynamic insights into therapeutic mechanisms [83]. The application of advanced imaging techniques and spatial transcriptomics will further enhance our ability to understand cellular interactions and drug distribution within organoids.

Organoid-based pharmacogenomics represents a transformative approach for understanding PBI and developing personalized therapeutic strategies. The ability to model human-specific aspects of brain development and injury in three-dimensional systems, combined with single-cell resolution profiling and patient-specific modeling, provides unique opportunities for precision medicine in neonatal neuroprotection [67,77,85]. While significant challenges remain, particularly regarding genetic diversity, physiological relevance, and clinical translation, ongoing technological advances and collaborative efforts are steadily addressing these limitations. As organoid technology continues to mature and integrate with other cutting-edge approaches, it holds great promise for identifying the most effective therapeutic targets and personalizing treatment strategies for individual patients with PBI. An overview of the literature on pharmacogenomic insights derived from organoid-based models of brain injury and their therapeutic implications is provided in Table 1.

## 7. Integration with Existing Therapies, Challenges, and Future Perspectives in Brain Organoid-Guided Therapies for PBI Treatment

### 7.1. Integration of Organoids with Pharmacological Neuroprotection

One of the most promising applications of brain organoids in perinatal injury research is their use as platforms for testing how pharmacological agents can be combined with cellular therapies. This study provided important proof of concept that organoid models can identify synergistic effects between pharmacological agents and endogenous repair mechanisms. The application of neuroprotective compounds to organoids before or during modeling of perinatal injury has revealed cell type-specific protective effects that would be difficult to detect in bulk tissue analysis. Shou and colleagues reviewed how various pharmacological interventions affect different stages of neuronal development in organoid systems, emphasizing the importance of timing in neuroprotective strategies [97]. These findings have important implications for clinical translation, as they suggest that therapeutic windows for different agents may vary depending on the developmental stage and cellular composition of injured tissue.

### 7.2. Bioengineering Approaches to Enhance Organoid Maturation and Integration

A major advancement in organoid-guided therapies has been the development of bioengineering strategies to enhance both in vitro maturation and post-transplantation integration. Cho and colleagues [98] developed a microfluidic device incorporating brain extracellular matrix components that significantly promotes structural and functional maturation of human brain organoids. This approach addresses one of the fundamental limitations of traditional organoid culture which is the lack of physiological microenvironmental cues that drive maturation. An innovative approach to overcoming these limitations is electrical stimulation that has emerged as particularly promising intervention for both organoid maturation and transplantation success. Li and colleagues [99] demonstrated that pre-treatment of brain organoids with electrical signals enhances their maturation in vitro and dramatically improves their integration when transplanted into injured brain tissue. Electrically stimulated organoids showed increased viability, enhanced synaptic activity, and better structural integration with host neural circuits compared to non-stimulated controls. This bioengineering approach represents a practical strategy that could be implemented clinically to prepare organoids for transplantation [99]. An approach addressing issues of spatiotemporal dynamics was demonstrated by Sağlam-Metiner et al. [100], who showed that mechanical stimulation and controlled media flow increase cellular diversity and neuronal function in cerebral organoids. These studies collectively indicate that recreating aspects of the dynamic in vivo environment is crucial for generating therapeutically relevant organoids.

### 7.3. Organoid Transplantation and Functional Integration

Direct transplantation of brain organoids into injured tissue represents a regenerative medicine approach that has shown considerable promise in preclinical models. Early work by Daviaud and colleagues [101] demonstrated that transplanted human cerebral organoids can vascularize and incorporate into mouse cortex, establishing a foundation for therapeutic applications. Subsequent studies have shown that these transplanted organoids are not only passive grafts but actively integrate into host neural circuits. Dong and colleagues [102] demonstrated that human cerebral organoids can establish subcortical projections in the mouse brain after transplantation, suggesting capacity for anatomical integration across brain regions. Other researchers extended these findings by showing axonal extensions along corticospinal tracts from transplanted organoids, indicating potential for long-range connectivity [103]. These structural integration studies are critical for understanding whether transplanted organoids can participate in functional neural networks. More recently, compelling evidence for structural and functional integration of human forebrain organoids with the injured adult rat visual system was provided [104]. This study showed that transplanted organoids not only survive and grow but also respond to visual stimuli and form functional connections with host neurons. The development of detailed protocols for organoid transplantation has further facilitated reproducibility and standardization of these approaches [105]. Cao and colleagues showed that cerebral organoid transplantation can repair infarcted cortex and restore impaired function after stroke, providing evidence of therapeutic efficacy in a clinically relevant injury model [106]. Other research demonstrated how organoid age affects post-transplant growth, finding that younger organoids show better integration than more mature ones [107]. These findings have practical implications for optimizing transplantation strategies.

### 7.4. Technical Challenges in Organoid-Based Therapies

Despite remarkable progress, several technical challenges must be addressed before organoid-guided therapies can be widely implemented clinically. Andrews and Kriegstein [108] provided a comprehensive overview of fundamental challenges in organoid research, including issues with maturation, cellular composition, and reproducibility. These challenges are particularly acute when considering therapeutic applications where consistency and safety are pivotal. One major limitation is the incomplete maturation of organoid-derived neurons and glia. Qian and colleagues [109] discussed how brain organoids, despite their sophistication, typically arrest at fetal-like developmental stages and fail to achieve full adult maturation. This limitation affects both their utility as disease models and their potential as cell replacement therapies, since immature cells may not integrate properly or could even pose tumorigenic risks [109]. Another significant challenge is lack of certain cell types. Most organoids lack mature oligodendrocytes, which are critical for myelination and whose dysfunction plays important role in PBI [110]. Similarly, the absence or immaturity of microglia in most organoid systems limits their ability to model neuroinflammatory components of injury [111]. Significant challenges for developing standardized therapeutic products are Batch-to-batch variability which is a persistent problem that affects reproducibility. That problem was discussed in work which showed how functional recovery through plastic adaptation of organoid grafts in cortical tissue can vary substantially depending on organoid preparation methods and donor cell lines [112].

### 7.5. Translational Barriers and Clinical Considerations

The translation of organoid-based therapies from laboratory to clinic faces numerous barriers beyond technical challenges. Bellotti and colleagues [93] discussed how organoids and chimeras require careful evaluation of safety, efficacy, and ethical considerations before clinical implementation. One critical translational challenge is the need for standardized protocols that can be implemented across different laboratories and clinical centers. Wang and colleagues [113] reviewed transplantation strategies to enhance maturity and cellular complexity in brain organoids, emphasizing the importance of reproducible methods. Without standardization, it will be difficult to conduct multi-center clinical trials or establish consistent manufacturing procedures. The development of organoid biobanks represents a potential solution for enabling rapid clinical deployment. Zhao et al. [114] discussed how emerging brain organoid biobanks could provide ready to use therapeutic products for acute injury settings where time is critical. However, developing such biobanks requires substantial infrastructure and raises questions about immunological compatibility and quality control. Also, ethical landscape surrounding organoid-based therapies is notably complex. Ajongbolo and Langhans [82] examined ethical frameworks applicable to brain organoids and assembloids, emphasizing critical issues such as the possibility of consciousness emergence, requirements for donor cell consent, and ethical boundaries for human brain tissue utilization. Resolution of these ethical dilemmas necessitates thorough engagement with diverse stakeholders and formulation of suitable regulatory guidelines.

### 7.6. Integration of Advanced Technologies

The integration of organoid platforms with other novel technologies promises to accelerate therapeutic development. Organoid-on-chip systems combine the biological complexity of organoids with the precision control of microfluidic devices [83]. These platforms enable high-throughput drug screening and more precise modeling of physiological parameters, which could facilitate optimization of combination therapies. Interfacing brain organoids with precision medicine and machine learning approaches can enhance therapeutic development. Such algorithms can analyze complex datasets from organoid experiments to identify patterns and predict therapeutic responses, potentially accelerating the discovery of effective treatment strategies [90].

Bioengineering tools for next generation neural organoids, including advanced biomaterials, biosensors, and automated culture systems, will be essential for successful clinical translation. These integrated technological approaches address fundamental limitations in current organoid systems while simultaneously enabling scalable production necessary for therapeutic applications [115].

### 7.7. Enhancing Transplantation Success Through Biological Factors

**Figure 2:**
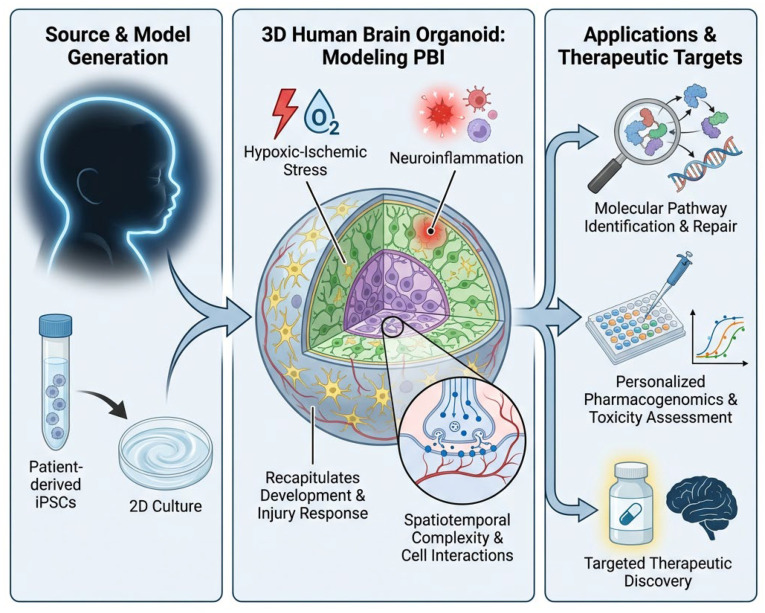
Schematic overview of brain organoids in perinatal brain injury (PBI) research. The figure illustrates the generation of 3D human brain organoids from patient-derived iPSCs (**left panel**), their recapitulation of spatiotemporal cellular complexity including neurons (green), glia (yellow), progenitors (purple), and synapses/connections (blue) (**central panel**), and applications in modeling hypoxic–ischemic stress, neuroinflammation, and injury responses (**right panel**). Organoids enable molecular pathway identification, repair mechanisms, pharmacogenomics, toxicity assessment, and targeted therapeutic discovery compared to traditional 2D cultures. Generated using GPAI—AI STEM Visualizer (GPAI pro model).

Beyond bioengineering approaches, biological factors can enhance organoid transplantation success. Progranulin, a growth factor involved in neurodevelopment and neuroinflammation, significantly enhances the engraftment of transplanted human iPSC-derived cerebral neurons [116]. Pre-treating organoids with such factors before transplantation could improve their survival and integration. Dispersing human neural stem cells in artificial extracellular matrix allows formation of cerebral organoids when incorporated in vivo [117]. This approach suggests that the transplantation strategy itself, including the use of supportive scaffolds can influence organoid integration and therapeutic efficacy. Pirrotte and colleagues [118] conducted single-cell analysis of neural stem cells and their extracellular vesicles, revealing distinct progenitor populations with neurogenic potential. Understanding the heterogeneity within organoid-derived cell populations and harnessing specific progenitor types could enable more targeted therapeutic applications. The process of brain organoid generation from induced pluripotent stem cells (iPSCs), along with the induction of various pathological conditions to investigate novel therapeutic approaches, is visually depicted in Figure 2.

## 8. Future Perspectives and Research Directions

Organoid models provide an ideal platform for testing to bridge developmental neurobiology and translational therapeutics, paving the way for targeted and individualized interventions in PBI, including different combination approaches before clinical implementation [119]. Brain organoid-guided therapies represent a transformative approach for treating PBI, offering both sophisticated modeling platforms to optimize existing treatments and potential cell replacement therapies. Integration of organoids with neuroprotective agents and bioengineering strategies has demonstrated considerable promise in preclinical studies [64,99,105,119]. However, significant technical challenges including incomplete maturation, limited vascularization, batch variability, and absence of immune components must be addressed before widespread clinical implementation [93,108,120]. Translational barriers related to standardization, safety assessment, ethical considerations, and regulatory frameworks also require careful attention [82,112,113]. Future research should focus on integrating immune and vascular components, establishing standardized protocols and biobanks, and leveraging advanced technologies such as organoid-on-chip platforms and machine learning [90,114,115]. With continued innovation and collaborative efforts across disciplines, organoid-guided therapies hold tremendous potential for improving outcomes in PBI.

The future of organoid-guided research and therapies for PBI will likely involve multiple converging approaches. Important aspect is bridging the gap from laboratory to clinic through systematic validation studies and development of GMP protocols. This will require collaborative efforts between researchers, clinicians, regulatory agencies, and industry partners [120]. Integration of immune components into organoid models represents an important future direction. Current organoids lack most immune cell types, limiting their ability to model neuroinflammation and test immunomodulatory therapies. Developing methods to incorporate microglia, astrocytes with appropriate reactive phenotypes, and potentially peripheral immune cells will enhance both the modeling and therapeutic potential of organoids. Standardization efforts must be prioritized to enable clinical translation, as well as quality control metrics, and characterization methods [110]. International consortia focused on organoid standardization are emerging and will be essential for establishing best practices. The application of organoids for personalized medicine approaches in PBI remains largely unexplored. Zhao and colleagues [114] discussed how emerging brain organoid technologies could enable individualized therapeutic strategies based on patient-specific cellular responses. This could involve generating organoids from individual patients to test therapeutic responses before treatment, similar to approaches being developed in oncology. Combination therapies represent another promising direction. Rather than using organoid transplantation alone, combining cellular therapy with pharmacological neuroprotection, growth factors, and rehabilitation strategies may provide synergistic benefits.

## 9. Conclusions

Perinatal hypoxia/ischemia remains a major unmet clinical challenge due to its complex and multifactorial pathophysiology and the limited efficacy of current therapies. Human brain organoids represent a powerful and physiologically relevant platform to model key cellular and molecular mechanisms of PBI, overcoming many limitations of traditional experimental systems. By enabling the study of injury responses, repair processes, and patient-specific drug effects, organoids bridge developmental neurobiology and translational research. Emerging evidence supports their utility in guiding neuroprotective, cell-based, and combination therapeutic strategies prior to clinical implementation. Nevertheless, significant hurdles (including incomplete maturation, lack of vascular and immune components, variability, and ethical and regulatory concerns) must be addressed to enable clinical translation. Future advances integrating bioengineering, immune modeling, standardized protocols, and personalized approaches are essential. With continued interdisciplinary collaboration, organoid-guided research holds substantial promise for advancing targeted, safe, and individualized therapies to improve outcomes in PBI.

## Figures and Tables

**Table 1:** Summary of key literature on pharmacogenomic insights derived from organoid-based models of brain injury or pathology and their implications for personalized therapeutic targeting.

Authors (Year)	Study Type	Main Thesis	Key Conclusion
Paşca et al. (2019) [56]	Experimental (in vitro, cortical spheroids)	Human 3D cortical spheroids model hypoxic brain injury of prematurity	Pharmacological modulation of UPR prevents hypoxia-induced loss of intermediate progenitors; validates organoids as a platform for personalized PBI drug testing.
Boisvert et al. (2019) [64]	Experimental (in vitro, human brain organoids)	Minocycline, a tetracycline antibiotic with anti-inflammatory properties, mitigates the effects of neonatal hypoxic insult on human brain organoids.	Organoids can identify clinically relevant therapeutic effects and validate neuroprotective agents.
Yi et al. (2025) [67]	Experimental (single-cell transcriptomics, vascularized organoids)	Single-cell transcriptomics of vascularized human brain organoids reveals lineage-specific stress adaptation mechanisms in fetal hypoxia-reoxygenation injury.	Distinct vulnerability patterns identified: developmental arrest in astrocyte precursors and neurogenic collapse in GABAergic neurons.
Li et al. (2025) [68]	Experimental (vascularized cerebral organoids)	Neural responses to hypoxic injury characterized in vascularized cerebral organoid models with improved physiological relevance compared to non-vascularized systems.	Vascularization enhances modeling of drug delivery and neurovascular interactions central to PBI pathophysiology.
Shin et al. (2024) [71]	Experimental (spinal cord organoids, necrotic core-free model)	Establishment and validation of a necrotic core-free spinal cord organoid model for fetal neural ischemia, enabling detailed cellular analysis without confounding massive cell death.	Model allows precise cell-level analysis of hypoxic–ischemic responses.
Kim et al. (2021) [80]	Experimental (3D human neural organoids)	3D human neural organoids model hypoxic brain injury, replicating key cellular responses to oxygen deprivation including effects of ISRIB and rapamycin.	Multiple neuroprotective compounds (ISRIB, rapamycin) validated in a human organoid system.
Daviaud et al. (2019) [63]	Experimental (cerebral organoids, prenatal hypoxia)	Distinct vulnerability and resilience profiles identified among human neuroprogenitor subtypes in a cerebral organoid model of prenatal hypoxic injury.	Cell-type-specific therapeutic targets identified for protecting the most vulnerable progenitor populations.
Jacob et al. (2019) [86]	Experimental (patient-derived GBM organoids, biobank)	Development of a patient-derived glioblastoma organoid biobank that recapitulates inter- and intra-tumoral heterogeneity for personalized drug testing.	Proof of concept for patient-specific organoid biobanks in precision medicine.
Peng et al. (2025) [87]	Clinical/translational (individualized patient tumor organoids)	Individualized patient tumor organoids faithfully preserve the human brain tumor ecosystem and predict patient response to therapy before clinical administration.	Strongest clinical proof of concept for personalized organoid-based medicine.
Wang et al. (2025) [88]	Experimental (phenotypic organoid atlas and biobank)	A phenotypic brain organoid atlas and biobank for neurodevelopmental disorders demonstrates the feasibility of large-scale personalized medicine initiatives based on organoids.	Scalability of organoid biobanks for personalized medicine confirmed.
Antón-Bolaños et al. (2024) [89]	Experimental (Chimeroids, multi-donor organoids)	Chimeroids—organoids generated from multiple donors—reveal individual susceptibility to neurotoxic triggers while controlling for technical variability.	Novel tool for assessing genetic factors influencing individual susceptibility to brain injury.
Bruno et al. (2025) [91]	Experimental (Raman spectroscopy + machine learning)	Label-free detection of biochemical changes during cortical organoid maturation using Raman spectroscopy combined with machine learning algorithms.	Advanced non-invasive analytical methods can extract pharmacological information from organoids without interference with organoid biology.
Zhu et al. (2024) [96]	Experimental (multi-omics, stroke organoids platform)	A stroke organoids-multiomics platform integrating transcriptomics, proteomics, and metabolomics to study injury mechanisms and drug responses comprehensively.	Integrated multi-omics approach provides comprehensive molecular profiles of injury and therapeutic responses.
Bellotti et al. (2024) [93]	Review (organoids, chimeroids, TBI)	Critical review of organoids and chimeroids in traumatic brain injury research	Limited genetic diversity across most organoid studies restricts generalizability; diverse biobanks are essential for valid pharmacogenomic variability assessment.
Hazra M. (2022) [94]	Systematic review and meta-analysis	Systematic review of pharmacogenomic mechanisms studied via brain organoids	A major gap exists between organoid-based pharmacogenomic discoveries and clinical translation; collaborative efforts among researchers, clinicians, and regulatory bodies are required.
Castiglione et al. (2022) [83]	Review (organoid-on-chip, microfluidics)	Organoid-on-chip technologies combine biological complexity of organoids with microfluidic precision, enabling high-throughput pharmacological studies with controlled microenvironmental conditions.	Microfluidic organoid platforms enhance drug screening capabilities by enabling precise oxygen gradients, localized insult simulation, and high-throughput pharmacological profiling.

## Data Availability

No new data were created or analyzed in this study.

## Abbreviations

The following abbreviations are used in this manuscript:

2D	Two-dimensional
3D	Three-dimensional
ATP	Adenosine triphosphate
BBB	Blood–brain barrier
BPD	Bronchopulmonary dysplasia
CP	Cerebral palsy
CRISPR	Clustered Regularly Interspaced Short Palindromic Repeats
DAMPs	Danger-associated molecular patterns
DNA	Deoxyribonucleic acid
EVs	Extracellular vesicles
EXs	Exosomes
GMP	Good Manufacturing Practice
HIE	Hypoxic–ischemic encephalopathy
HIF-1α	Hypoxia-inducible factor 1-alpha
IL-1β	Interleukin-1 beta
ISRIB	Integrated Stress Response Inhibitor
LPS	Lipopolysaccharide
MVs	Microvesicles
OGD	Oxygen-glucose deprivation
PBI	Perinatal brain injury
RNA	Ribonucleic acid
ROS	Reactive oxygen species
SC-EVs	Stem cell-derived extracellular vesicles
TH	Therapeutic hypothermia
TNFα	Tumor necrosis factor alpha
hAECs	Human amnion epithelial cells
iPSCs	Induced pluripotent stem cells
